# Nature-Adventure based experiential methods for enhancing psychotherapeutic efficacy

**DOI:** 10.1192/j.eurpsy.2024.156

**Published:** 2024-08-27

**Authors:** E. Kenézlői, N. Rákár-Szabó, D. Szabó, E. Lévay, G. Szabó, A. Szegő, B. Hajduska-Dér, I. Seres, I. Császár, J. Hordósi, K. Zseli, J. Réthelyi, Z. S. Unoka

**Affiliations:** ^1^Psychiatry and Psychotherapy, Semmelweis University; ^2^Kétté Alapítvány, Budapest, Hungary

## Abstract

**Introduction:**

A complex, Nature-, and Adventure Therapy - integrated Schema Therapeutic program (N-ABST) and a related efficacy study was launched in 2022 April at the Psychotherapy Department, at Semmelweis University. The participants had the opportunity of having outdoor, experience based group processes – seven full days in a month - in addition to the classic Schema Therapy (ST) sessions. According to the study design, 4-week long traditional thematic ST programs and 4-week long N-ABST programs were taken place alternately.

**Objectives:**

Our aim was to compare the efficacy in a randomized, controlled design, short and medium terms. The participants of the programs and thus the target group of the research were adults, diagnosed mainly with Borderline Personality Disorder, inpatients in psychiatry.

**Methods:**

This methodological innovation also meant the integration of two therapeutic teams in practice. When establishing the collaboration, we put emphasis on finding common points and understanding how N-AT contributes to schema therapy goals. During our joint work, it became clear that the elemental need for contact with nature enriched the schema therapy approach with a new basic need that was not included in it before. Measurements were taken before the start of the entire program and at the end of the 4-week cycle. Preliminary results are presented based on the Personality Inventory for DSM-5 - Hungarian Short Form (PID-5-HSF), and the Derogatis Symptom Checklist (SCL90).

**Results:**

In the N-ABST group (n=23) the PID5 “Dysinhibition” scale (p < .01, Cohen’s d = .636), and the “Negative Affectivity” scale (p < .05, Cohen’s d = .388) showed significantly lower scores after therapy. In the case of the “Detachment” we have found a tendency to decrease after the therapy. Regarding the comparison of the effectiveness of N-ABST and classical Schema Therapy - with the current state of analysis - there was a significant difference in the PID5 values for “Suspiciousness” and “Manipulativeness”. The former characteristic was reduced to a greater extent by the schema therapy, and the latter by the N-ABST therapy. Based on the SCL90, the N-ABST program resulted in a significant symptom reduction measured by the following subscales: somatization, obsessive compulsive, interpersonal sensitivity, depression, phobia. Global symptom severity also decreased significantly (p < .05, Cohen’s d = .588).

**Conclusions:**

According to our results, Nature- Adventure Therapy enhanced Schema Therapy seems to be an innovative and efficient method in the psychotherapy of personality disorders. Besides the effectiveness, there is a great challenge to design programs that are sustainable and therefore serves therapy long term as well.

This study was supported by the National Research, Development and Innovation Office grant K 129195.

**Disclosure of Interest:**

None Declared

